# Progression of inflammation during immunodeficient mouse skeletal muscle regeneration

**DOI:** 10.1007/s10974-015-9433-1

**Published:** 2015-11-27

**Authors:** Iwona Grabowska, Magdalena A. Mazur, K. Kowalski, A. Helinska, Jerzy Moraczewski, Władysława Stremińska, Grażyna Hoser, Jerzy Kawiak, Maria A. Ciemerych, Edyta Brzoska

**Affiliations:** Department of Cytology, Faculty of Biology, University of Warsaw, Miecznikowa 1 St., 02-096 Warsaw, Poland; Laboratory of Flow Cytometry, Medical Center of Postgraduate Education, Marymoncka 99/103 St., 01-813 Warsaw, Poland

**Keywords:** Inflammation, Muscle regeneration, Immunodeficiency

## Abstract

The skeletal muscle injury triggers the inflammatory response which is crucial for damaged muscle fiber degradation and satellite cell activation. Immunodeficient mice are often used as a model to study the myogenic potential of transplanted human stem cells. Therefore, it is crucial to elucidate whether such model truly reflects processes occurring under physiological conditions. To answer this question we compared skeletal muscle regeneration of BALB/c, i.e. animals producing all types of inflammatory cells, and SCID mice. Results of our study documented that initial stages of muscles regeneration in both strains of mice were comparable. However, lower number of mononucleated cells was noticed in regenerating SCID mouse muscles. Significant differences in the number of CD14-/CD45+ and CD14+/CD45+ cells between BALB/c and SCID muscles were also observed. In addition, we found important differences in M1 and M2 macrophage levels of BALB/c and SCID mouse muscles identified by CD68 and CD163 markers. Thus, our data show that differences in inflammatory response during muscle regeneration, were not translated into significant modifications in muscle regeneration.

## Introduction

Skeletal muscle regeneration relies on the differentiation of tissue specific stem cells, i.e. satellite cells (Montarras et al. 2013; Scharner and Zammit 2011). The activation of satellite cells, occurring in response to the muscle injury, is manifested by the resumption of the cell cycle and differentiation into myoblasts [reviewed in (Ciemerych et al. 2011)]. Satellite cells start to proliferate approximately 18–24 h after the injury. At days 3 and 4 of regeneration the proliferation of mouse satellite cells is the most intense and it decreases during next 7–10 days (Grounds and McGeachie 1987). Simultaneously, at day 4 of regeneration satellite cells-derived myoblasts fuse into myotubes and subsequently into muscle fibers which formation is almost completed within 7–10 days after the injury (Robertson et al. 1990). Newly formed myotubes either fuse with the ends of the existing myofibers or form new myofibers and finally the innervation is restored (Robertson et al. 1993). Within 14 days after the injury full restoration of muscle architecture and function is completed (Robertson et al. 1993).

Importantly, muscle injury triggers inflammatory response which plays a crucial role both in muscle fiber degeneration as well as activation and differentiation of satellite cells [reviewed in (Brzoska et al. 2011)]. In response to local vascular damage, which is inevitable during extensive muscle injury, and signals released by degenerating muscle fibers, the inflammatory cells become attracted from the bloodstream and infiltrate the injured muscle (Kharraz et al. 2013). At first, inflammatory cells infiltrate the injured area and phagocyte the damaged tissue. Next, inflammatory cells release growth factors and cytokines that activate the migration and proliferation of satellite cells, and finally, produce enzymes to modify the extracellular matrix (ECM) (Kharraz et al. 2013). The first population of inflammatory cells that infiltrate the injured muscle are neutrophils which arrive within 2 h post injury. Later their number decreases and neutrophils become undetectable 3–4 days later (Tidball 2005; Tidball and Villalta 2010). Neutrophils phagocytose damaged fibers, release the reactive oxygen species (ROS), as well as proteases and cytokines to attract monocytes (Kharraz et al. 2013; Lockhart and Brooks 2008; Pizza et al. 2001).

Muscle damage induces migrating and also resident monocytes to differentiate into macrophages. Pro-inflammatory macrophages are called M1 since they are the first population of macrophages observed after the muscle injury. They phagocytose necrotic muscle fibers, participate in antigen presentation, produce pro-inflammatory cytokines (e.g. TNFα, IL-1β), and express inducible nitric oxide synthase which allows to metabolize l-arginine to produce NO (Arnold et al. 2007). Second population of macrophages participating in skeletal muscle repair are M2 macrophages presenting anti-inflammatory activity by expressing anti-inflammatory cytokines, such as IL-10 and TGFβ (Villalta et al. 2009). Pro-inflammatory M1 cells were shown to positively influence myoblast proliferation and repress myoblast differentiation (Arnold et al. 2007). On the other hand anti-inflammatory cytokines produced by M2 cells promote myogenesis, enhance angiogenesis, and stimulate deposition of ECM components (Deng et al. 2012; Zhang et al. 2013). ECM is mainly produced by muscle resident fibroblasts that migrate to the site of injury immediately after muscle damage (Mann et al. 2011; Serrano et al. 2011). Proper remodeling of ECM in damaged muscle is critical for the reconstruction of scaffold structures required for the correct alignment of newly formed muscle fibers, and thus, for the regaining of muscle function. Incorrect ECM deposition may lead to the fibrosis of injured muscle.

Interestingly, the role of T cells and mast cells seems to be rather limited in muscle regeneration (Kharraz et al. 2013). Thus, 8 h after the injury the mast cells accumulate and release many pro-inflammatory cytokines (e.g. TNFα, IL-1, IL-6). On the other hand, T cell–secreted cytokines that play a role in maintaining activation of macrophages and produce anti-fibrotic (IFNγ, TNFα, IL-2, IL-12) and pro-fibrotic cytokines (IL-4, IL-5, IL-6, IL-13). Knowing that, we asked whether there are any differences in skeletal muscle regeneration of severe combined immunodeficiency (SCID) mice, i.e. animals that are characterized by the lack of the humoral and cell-mediated immunity due to the absence of mature T and B lymphocytes (Renz et al. 1996). SCID mice were derived from BALB/c C.B-17 mice (Bosma et al. 1983). They accept xenogeneic grafts and for this reason they are widely used as a model in transplantation studies (Vormoor et al. 1994). However, they are not a perfect model since they are characterized by the presence of NK cells. Thus, cyclophosphamide treatment is necessary before xenogeneic cells implantation (Basch et al. 1997). Despite this “imperfection” SCID mice are often used as a model to study myogenic potential of stem cells, including human-derived stem cells (Brzoska et al. 2006; Dellavalle et al. 2007; Grabowska et al. 2012, 2013; Morosetti et al. 2010). In this study we compared BALB/c (control) and SCID mouse skeletal muscle regeneration and inflammatory process accompanying this process.

## Materials and methods

### Muscle injury

All experimental procedures involving animals were approved by the Institutional Review Boards of the Medical Centre of Postgraduate Education, Warsaw. BALB/c and SCID mice were bred and maintained under defined flora conditions at the Department of Clinical Cytology, Medical Center of Postgraduate Education, Warsaw. Breeding of mice was initiated from BALB/c C.B-17-Icr Hnd Hsd SCID animals obtained from Harlan Farms, UK, and animals were frequently controlled for the absence of B and T lymphocytes. Males 8–10 weeks of age were used for the experiments. Animals were anesthetized with pentobarbital sodium salt (Sigma-Aldrich) by an intraperitoneal injection (30 mg/kg of body mass). The mouse gastrocnemius muscle was crushed mechanically as previously described (Brzoska et al. 2006). Treated animals were euthanized (days 0–7 and 30) and muscles were isolated, weighed, and processed for further analyses.

### Histochemistry

The isolated gastrocnemius muscles were frozen in isopentane cooled with liquid nitrogen, transferred to −80 °C, cut into 7 µm thick sections with a cryomicrotome, and stored at 4 °C. Sections were hydrated and fixed in 3 % PFA (paraformaldehyde) in PBS, for 10 min. Next, sections were stained with Harris hematoxylin–eosin Y. The slides were examined with the Eclipse TE 200 microscope (Nikon Instruments, Tokyo, Japan) equipped with ACT-1 software. Sections (3–10 per each variant) were analyzed using GIMP or ImageJ to evaluate the number of mononuclear cells, muscle fiber diameter and percentage of the tissue area occupied by the connective tissue.

### Cell isolation

A single cell suspension was obtained by treatment of gastrocnemius muscle fragments with 0.15 % pronase (Sigma-Aldrich), as described previously (Brzoska et al. 2006). Pronase is a mixture of proteases and it allows to isolate a more pure population of myogenic cells than collagenase or dispase (Danoviz and Yablonka-Reuveni 2012). Next, the cells were washed in PBS and either counted in a hemocytometer or processed for FACS analysis.

### FACS analysis

The FACSCalibur (Becton–Dickinson, San Jose, CA, USA) equipped with a 488-nm argon laser was used. Four data parameters were acquired and stored: FSC, SSC, fluorescence 1—FL1 (fluorescein isothiocyanate, FITC), and fluorescence 2—FL2 (phycoerythrin, PE). CellQuest application, version 1.2, was used for the analysis. Cytometer readings were controlled systematically by Becton–Dickinson-approved service. The isolated cells were fixed in a 70 % ethyl alcohol. Next, cells were washed with PBS and incubated in 2 % BSA in PBS followed by anti-CD14 and CD45 antibodies (rat anti-mouse CD45 FITC conjugated clone 30-F11, monoclonal rat anti-mouse CD14 PE conjugated clone rmC5-3, Becton–Dickinson Bioscience). Next, cells were washed in PBS, fixed in 0.5 % PFA in PBS, and subjected to FACS analysis.

### Immunocytochemistry

Gastrocnemius muscles isolated at day 7 of regeneration were frozen, cut into 7 µm thick sections, hydrated, and fixed in 3 % PFA in PBS, for 10 min. Unspecific antibody binding was blocked by the incubation of muscle sections in 3 % BSA in PBS at room temperature, for 30 min. Next, sections were incubated with antibody mix (dilution 1:50 in 3 % BSA in PBS) in a humidified chamber, at room temperature, for 1 h. Sections were incubated with FITC-conjugated rat monoclonal anti-mouse CD45 or PE-conjugated rat monoclonal anti-mouse CD14 antibodies (Becton–Dickinson Bioscience) or with primary antibodies: rabbit polyclonal anti-CD68 (Abcam) and mouse monoclonal anti-CD163 (AbD Serotec), and secondary antibodies anti-mouse IgG Alexa594 conjugated (Invitrogen) or anti-rabbit IgG Alexa488 conjugated (Invitrogen). Slides were rinsed with PBS and stained with DraQ5 (BioStatus) or chromomycin A3 (Sigma-Aldrich) to visualize chromatin, according to the manufacturer’s instructions. Finally, sections were analyzed using the confocal microscope Axiovert 100 M (Carl Zeiss Inc.) imaging system and the LSM 510 software (Carl Zeiss Inc.).

### Western blotting

Proteins were isolated from gastrocnemius muscles using cOmplete Lysis-M EDTA-free kit (Roche Applied Science). 25 µg of total protein lysate was denatured by boiling in Laemmli buffer and separated using SDS-Page electrophoresis and then, transferred to PVDF membrane (Roche Applied Science). Membranes were blocked with 5 % Blotto (BioRad)/TBS for 1 h and incubated with primary antibodies diluted 1:2000 in 5 % Blotto (BioRad)/TBS, at 4 °C, overnight, followed by secondary antibodies diluted 1:20,000, at room temperature, for 2 h. Protein bands were visualized using SuperSignal West Pico Chemiluminescent Substrate (Thermo Scientific) and exposed to chemiluminescence positive film (Amersham Hyperfilm ECL, GE Healthcare). The following primary antibodies were used: rabbit polyclonal anti-CD68 (Abcam), mouse monoclonal anti-CD163 (AbD Serotec), mouse anti-tubulin (Sigma Aldrich). Secondary antibodies used were: peroxidase-conjugate rabbit anti-mouse IgG (Sigma-Aldrich), peroxidase-conjugate goat anti-rabbit IgG (Sigma-Aldrich). Results were analyzed with Gel Doc™ XR+ System using Image Lab software (BioRad).

All experiments were performed 2–10 times (biological repeats). Results were shown as mean or median, and standard or mean deviations were presented. *t* test was performed for statistical analysis.

## Results

### Skeletal muscle regeneration of BALB/c and SCID mice

**Fig. 1:**
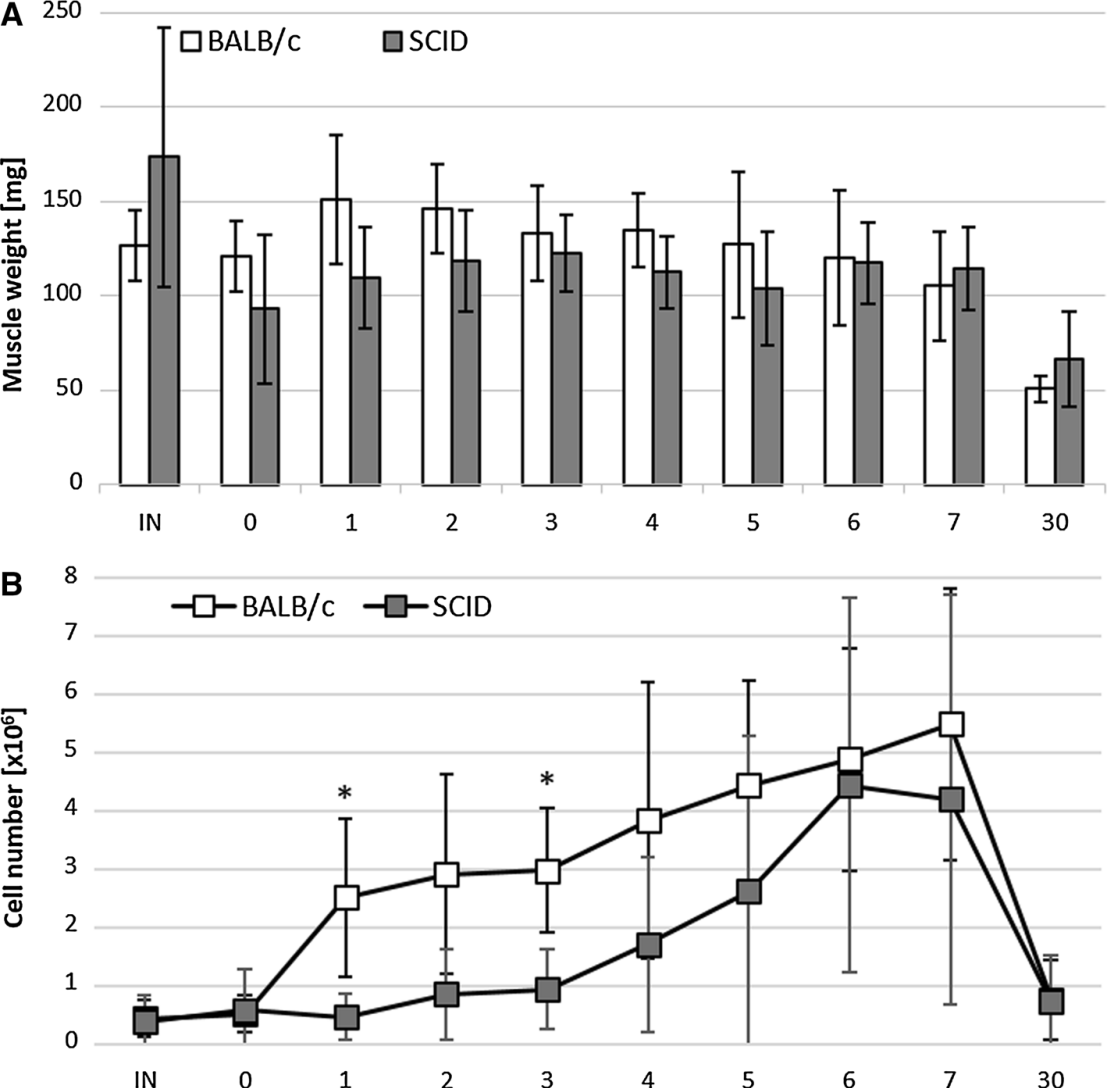
BALB/c and SCID mice gastrocnemius muscle regeneration. **a** The muscle weight. **b** The number of cells isolated from the muscles. Results were shown as means and standard deviations. Statistically significant differences were marked with *asterisk* (*t* test, p < 0,05). IN—intact muscle, day 0—1 h after injury, day 1—7 and 30 of regeneration

The regenerating gastrocnemius muscles of BALB/c and SCID mice were analyzed approximately 1 h (day 0) and at day 1–7, as well 30 days post injury. As a control we used intact muscles of both strains of mice. Results of this analysis showed that the mean mass of regenerating SCID mouse muscles (n = 5) was lower than that of BALB/c muscles (n = 3) (Fig. 1a). However, the differences between muscle mass of BALB/c and SCID mice were not statistically significant for either intact muscles or those analyzed at day 0–7 and day 30 of regeneration.

The number of mononucleated cells isolated from intact muscles of BALB/c (n = 4) and SCID (n = 2) mice was similar (Fig. 1b). During first days of regeneration (1–5) the number of cells isolated from the SCID mice muscles was lower as compared to BALB/c muscles (Fig. 1b). However, differences were statistically significant only at day 1 and 3 of regeneration (*t* test, p = 0.05 and 0.04, respectively).

**Fig. 2:**
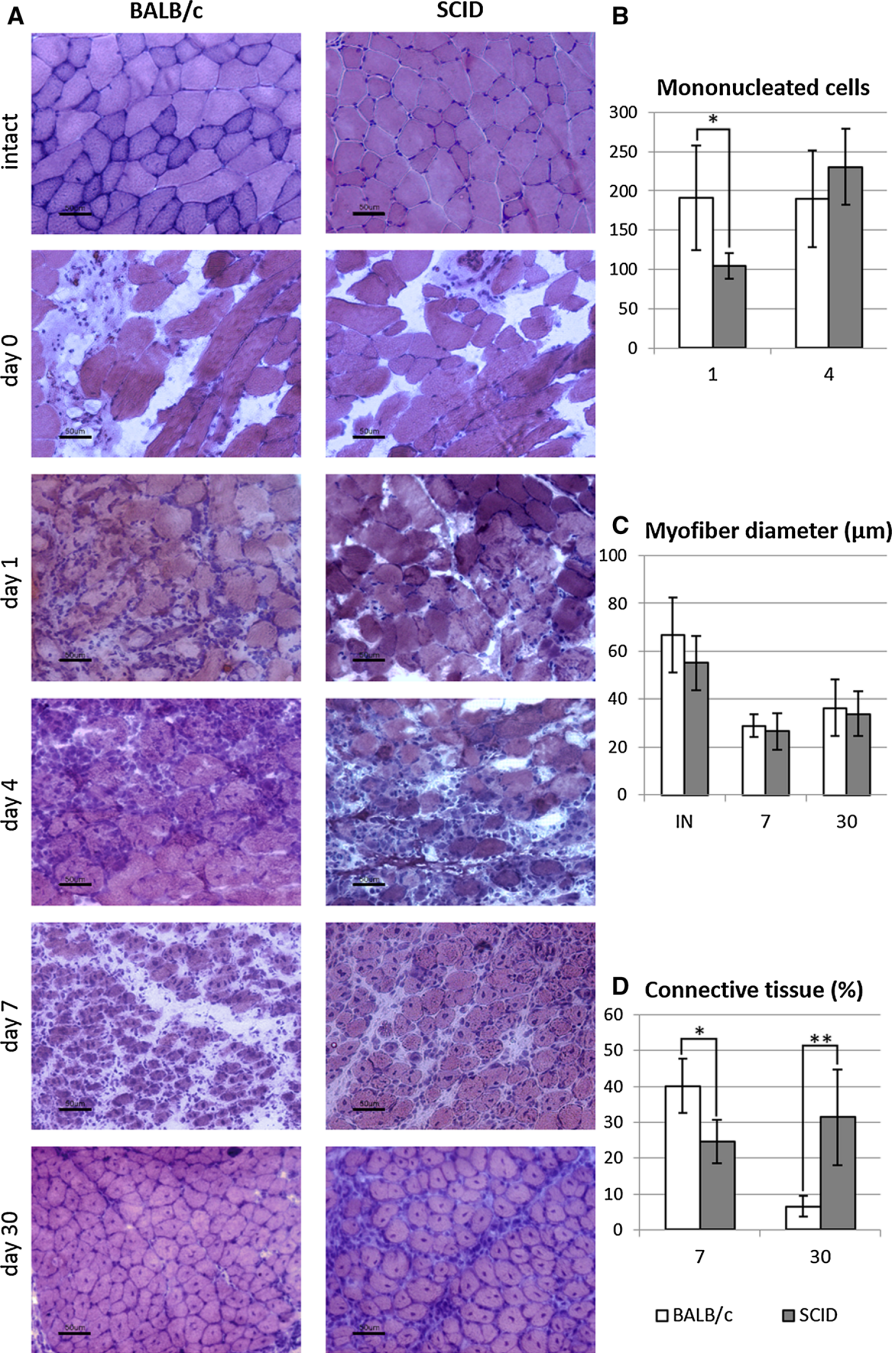
**a** Histological analysis of BALB/c and SCID mice gastrocnemius muscle regeneration. Harris hematoxylin-eosin staining. Scale bar = 50 µm. **b** The number of mononucleated cells present in the muscle at day 1 and 4 of regeneration. **c** The myofiber diameter in intact (IN) muscle and day 7 and 30 of regeneration. **d** The area of connective tissue at day 7 and 30 of BALB/c and SCID mice gastrocnemius muscle regeneration. Results were shown as means and standard deviations. Statistically significant differences were marked with *asterisk* (*t* test, p < 0,05)

Histological analysis of regenerating muscles did not showed significant differences between BALB/c and SCID mice muscles regeneration (Fig. 2a). At day 1 of regeneration the influx of mononuclear cells and degenerating muscle fibers were characteristic for muscles of both mouse strains. Moreover, more mononucleated cells were detected in BALB/c mice muscle at day 1 of regeneration. The number of mononucleated cells was significantly higher in BALB/c than in SCID mouse muscles (*t* test, p = 0.014, BALB/c mouse muscle n = 10, SCID mice muscles n = 5) (Fig. 2b). At day 4 post injury formation of small myotubes and new muscle fibers with centrally located nuclei were noted (Fig. 2a). The new muscle fibers, with centrally located nuclei and larger diameter, were observed at day 7. However, no statistically significant differences between BALB/c and SCID mice muscle fibers diameters at day 7 and 30 of regeneration were detected (*t* test, n = 3) (Fig. 2c). At day 30 the regeneration was completed, although, immature muscle fibers with centrally located nuclei were still detectable (Fig. 2b). Moreover, at day 30 of regeneration the lower level of fibrosis was observed in BALB/c mice muscles, as compared with SCID. The area of connective tissue was significantly lower in BALB/c than SCID mice muscles at day 30 of regeneration (*t* test, p = 0.006, BALB/c mice muscle n = 4, SCID mice muscles n = 7) (Fig. 2d).

### Participation of CD14+ and CD45+ cells in muscle regeneration

**Fig. 3:**
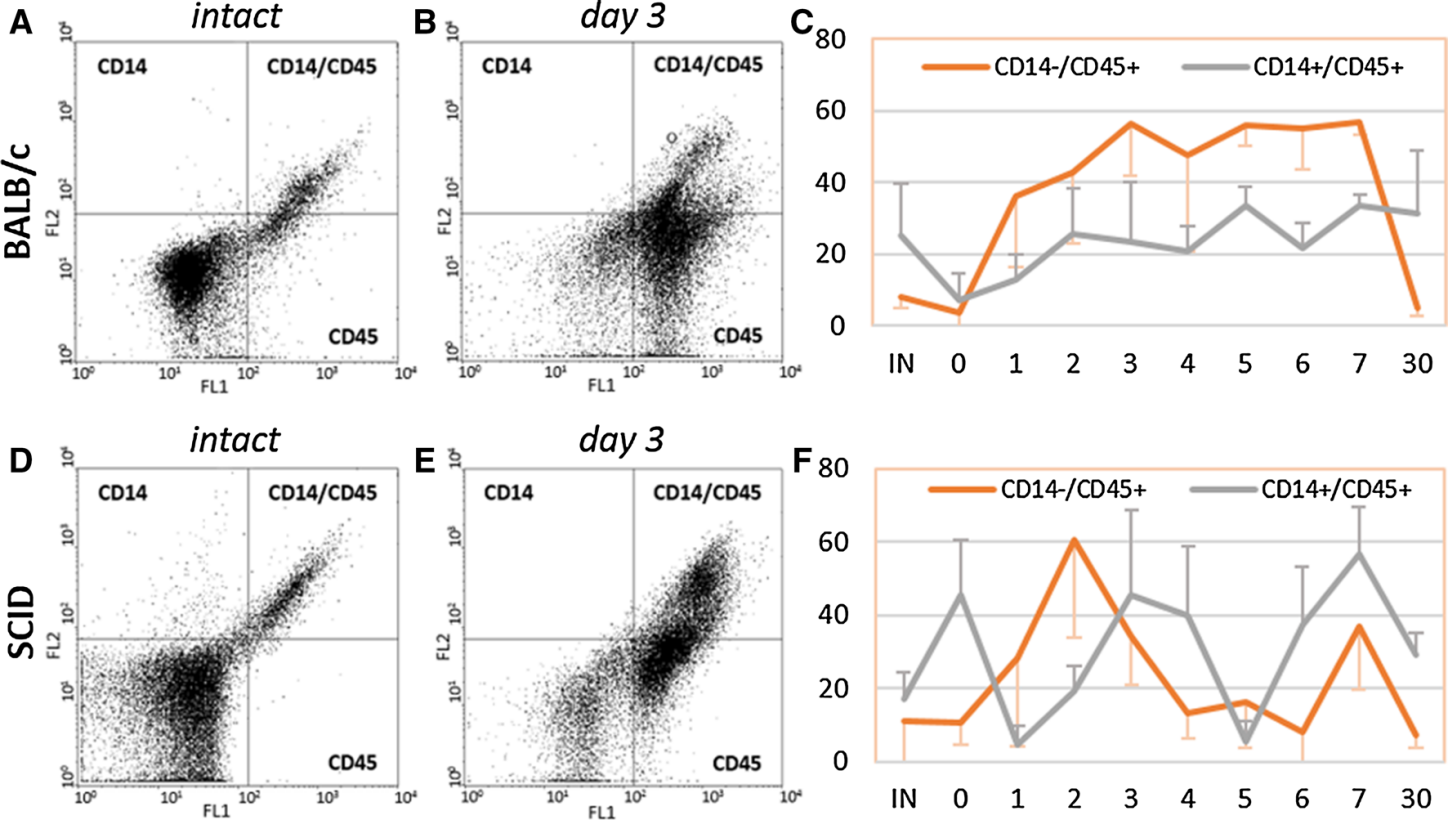
Analysis of CD14+/CD45+ and CD14−/CD45+ cells isolated from regenerating gastrocnemius muscle of BALB/c and SCID mice. **a** FACS analysis of cells isolated from intact BALB/c mouse muscle. **b** FACS analysis of cells isolated from BALB/c mouse muscle at day 3 of regeneration. **c** The proportion of CD14+/CD45+ and CD14−/CD45+ cells within the cells isolated from regenerating BALB/c mouse muscle. **d** FACS analysis of cells isolated from intact SCID mouse muscle. **e** FACS analysis of cells isolated from SCID mouse muscle at day 3 of regeneration. **f** The proportion of CD14+/CD45+ and CD14−/CD45+ cells within the cells isolated from regenerating SCID mouse muscle. Results were shown as medians and mean deviations. IN—intact muscle, day 0—1 h after injury, day 1—7 and 30 of regeneration

To follow the progress of inflammation in regenerating muscles of BALB/c and SCID mice we analyzed the number of macrophages (CD14+/CD45+) and hematopoietic cells (CD14−/CD45+ cells), i.e. granulocytes, T-cells, B-cells, thrombocytes, but not erythrocytes, in intact muscle at 1 h (day 0) and at day 1–7, as well as day 30 after injury (n = 3)(Fig. 3). The number of macrophages (CD14+/CD45+ cells) was similar in samples obtained from intact muscles of BALB/c and SCID mice. Analysis of cells isolated from BALB/c muscles showed that between days 1 and 7 post injury the number of CD14+/CD45+ cells slightly increased. In SCID mice muscles, analyzed 1 h after the injury, the number of CD14+/CD45+ cells was higher than in intact muscle. The number of CD14+/CD45+ cells was significantly higher in SCID mice muscle than BALB/c mice muscle at day 0 of regeneration (p = 0.05). Then, at day 1, it decreased and increased again at day 2, 3, and 4 of in regenerating SCID mouse muscles regeneration. At day 5 the decrease in CD14+/CD45+ cells number was observed, and it was significantly lower than in BALB/c mice muscle (p = 0.01). We noticed higher number of CD14+/CD45+ cells at day 6 and 7 in regenerating muscles of SCID mice. At day 30 of regeneration the number of CD14+/CD45+ cells was comparable with that observed in intact muscles. Thus, changes in macrophages level during SCID mice muscles regeneration were significant, while the changes in BALB/c mice muscles were less pronounced.

**Fig. 4:**
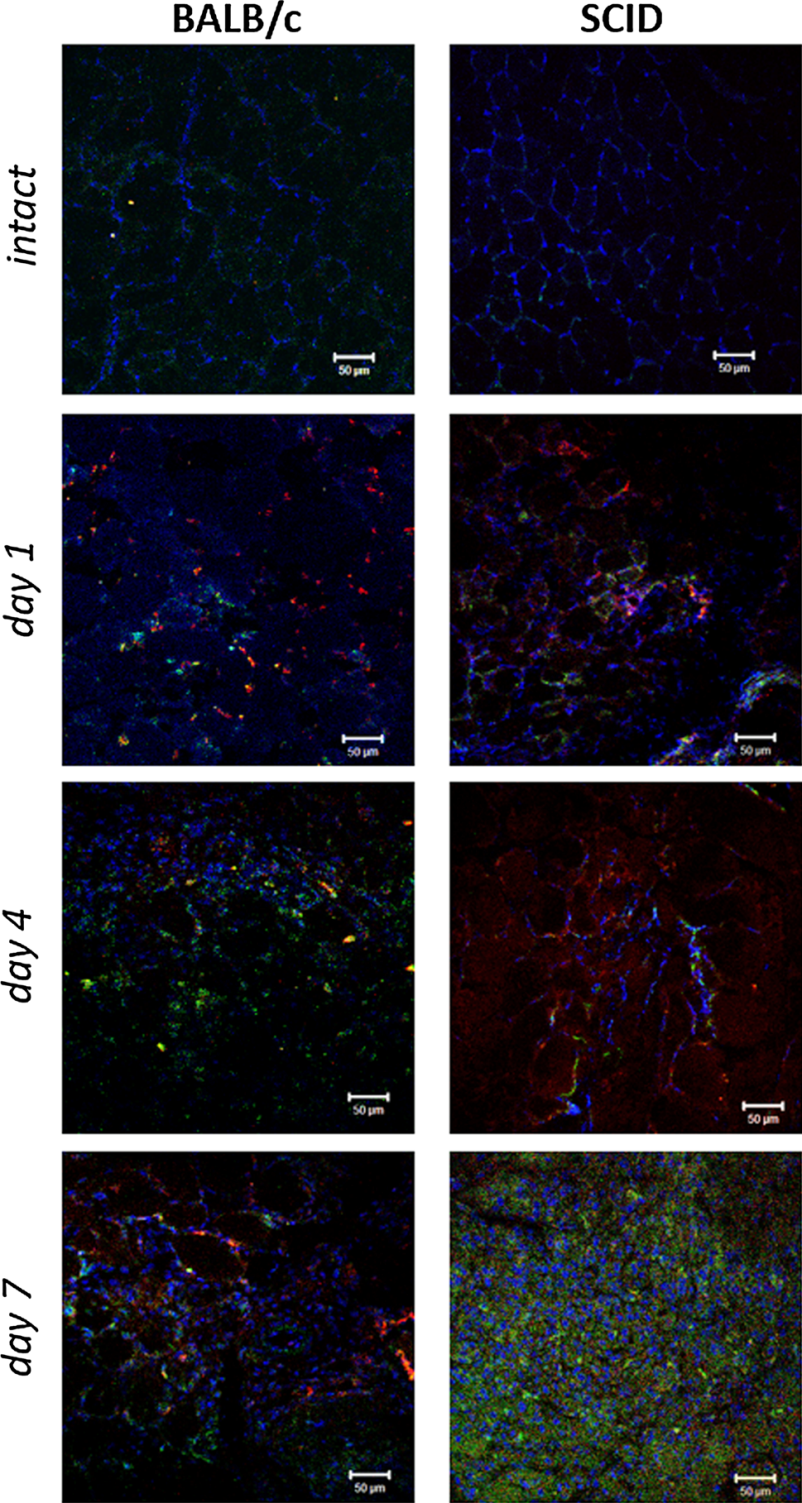
Immunolocalization of CD14+, CD45+, and CD14+/CD45+ cells in intact and regenerating BALB/c and SCID mice gastrocnemius muscles. Single CD14+/CD45+ cell were observed in BALB/c muscle. At day 1 of regeneration the influx of CD14+, CD45+ and CD14+/CD45+ cells were noticed both in BALB/c and SCID mice gastrocnemius muscles. A large number of CD45+ and some CD14+ and CD14+/CD45+ were present in BALB/c muscle at day 4 and 7 of regeneration. The SCID muscles were characterized first by decrease of inflammatory cells number (day 4) and then increase of their amount (day 7). CD14+ (*red*), CD45+ (*green*), and CD14+/CD45+ (*yellow*). Scale bar = 50 µm. (Color figure online)

The number of other hematopoietic cells, i.e. CD14−/CD45+ increased during first 24 h and the highest level of these cells was observed at day 3 of BALB/c mice muscle regeneration. The number of cells was high also at day 4–7 and decreased to the levels observed in intact muscles of BALB/c mice by 30 day after injury. In case of SCID mice the number of CD14−/CD45+ cells increased slower as compared to BALB/c mice muscles. The highest number of CD14−/CD45+ cells was noticed at day 2 of regeneration, then decreased and was significantly lower than in BALB/c mice muscles analyzed at day 5 of regeneration (p = 0.02). Subsequent slight peak of CD14−/CD45+ cells was observed in SCID mice muscles at day 7 of regeneration. Moreover, our FACS analysis were confirmed by immunolocalization of CD14+/CD45+ and CD14−/CD45+ cells during regeneration of BALB/c and SCID muscles (Fig. 4). Results of these analysis reflected almost precisely those obtained by FACS.

### The participation of M1 and M2 macrophages cells in muscle regeneration

**Fig. 5:**
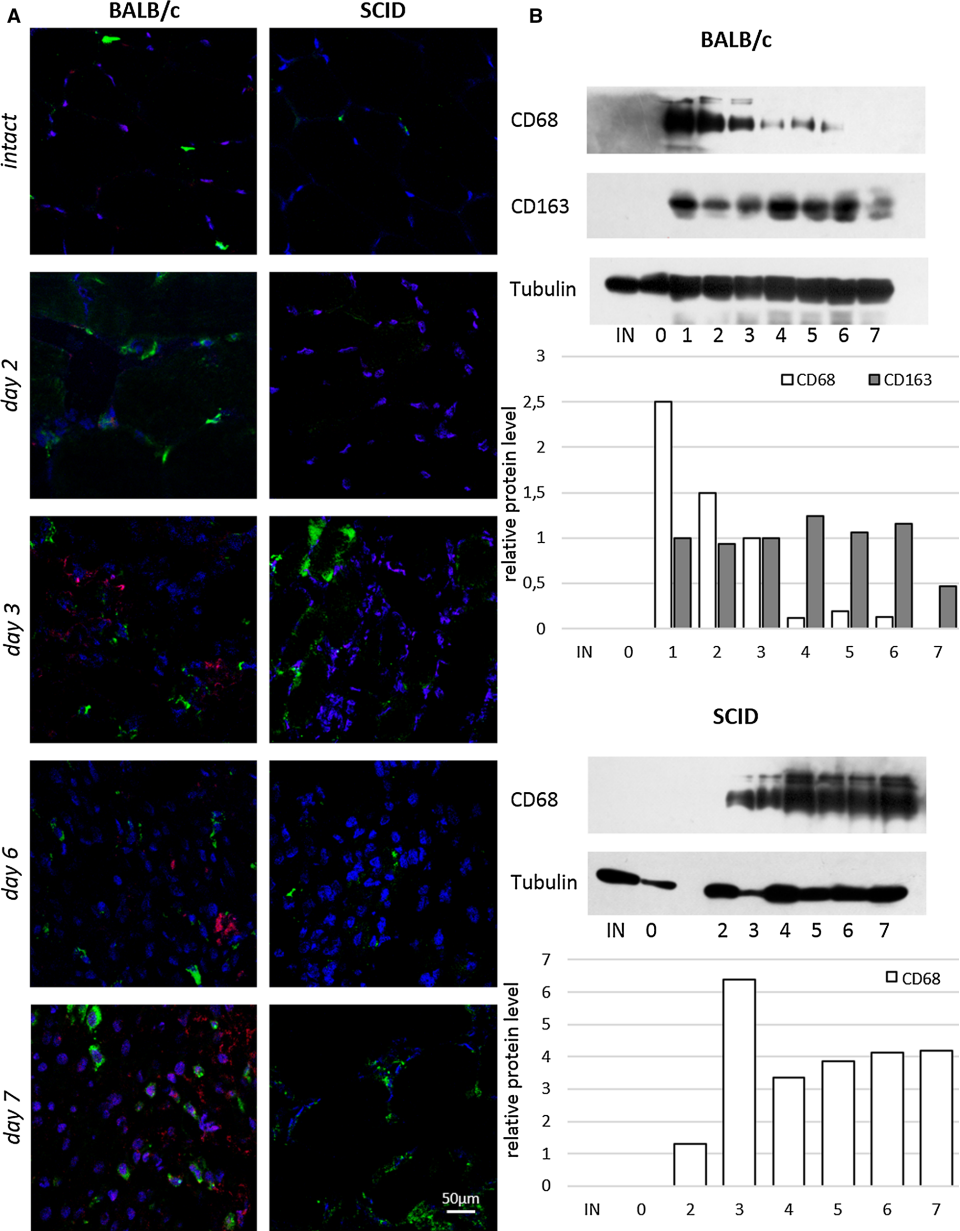
The M1 and M2 macrophages during regeneration of BALB/c and SCID mice muscles. **a** Immunolocalization of CD68+ (*green*) cells (M1 macrophages) and CD163+ (*red*) cells (M2 macrophages) in intact and regenerating BALB/c and SCID mice gastrocnemius muscles (day 2, 3, 6 and 7). Scale bar = 50 µm. *Blue*—nuclei. **b** The level of CD68, CD163, and tubulin protein in intact muscle (IN) and at day 0 (muscle injury)—7 of BALB/c and SCID mice gastrocnemius muscles regeneration. The *chart* presents relative optical densities of bands shown as a percentage of tubulin bands densities. (Color figure online)

Observed differences in the level of CD14+/CD45+ between BALB/c and SCID regenerating muscles prompted us to follow the M1 and M2 macrophage populations. Macrophages form heterogeneous population and could be divided into two groups—M1 and M2 (Barros et al. 2013; Rőszer 2015). M1 macrophages are characterized by a pro-inflammatory phenotype, while M2 macrophages play regulatory functions in tissue repair. In our study M1 macrophages were identified on the basis of CD68 expression and M2 macrophages on the basis of CD163 presence. In both SCID and BALB/c mice muscles, as shown directly by immunolocalization of CD68 marker (Fig. 5a), only few M1 macrophages expressing CD68 were detected in intact muscles. In BALB/c mice muscles, as shown by Western blotting technique (Fig. 5b), CD68 protein was detectable starting from day 1 of regeneration and its expression was the highest at day 1 and then decreased by day 7 of regeneration. In SCID mice muscles the level of CD68 was higher than in BALB/c mice muscles at day 3–7 of regeneration (Fig. 5b). The level of CD68 increased by day 3 of SCID mice muscle regeneration, then at day 4 decreased and did not changed until day 7. Interestingly, M2 macrophages were present in regenerating BALB/c mice muscles (Fig. 5a) but were not detected in SCID mice muscles as shown by immunolocalization (Fig. 5a) and Western blotting (Fig. 5b) of CD163. In BALB/c mice muscles the level of CD163 increased during regeneration starting from day 4 of regeneration (Fig. 5b). At day 7 of BALB/c mice muscles regeneration the level of CD163 decreased (Fig. 5b).

## Discussion

SCID mice are widely used in studies investigating myogenic potential of stem cells after transplantation into regenerating muscles (Arpke et al. 2013; Brzoska et al. 2006; Dellavalle et al. 2007; Grabowska et al. 2012, 2013; Morosetti et al. 2010). However, these mice could differ from other strains in the progression of muscle repair. In fact, differences in inflammatory and muscle regeneration processes were previously observed between mice of different genetic backgrounds (Lagrota-Candido et al. 2010). For example, regenerating muscles of athymic BALB/c nude (nu/nu) mice, lacking T lymphocytes, are characterized by higher collagen deposition than in muscles of C57BL/6 mice (Lagrota-Candido et al. 2010). It was observed that wild type BALB/c mice, i.e. producing T cells, are characterized by higher collagen deposition as compared to C57BL/6 mice (Lagrota-Candido et al. 2010). Thus, in this case fibrosis depends on the genetic background, not the lack of T cells. On the other hand, the absence of T and B lymphocytes in dysferlin deficient mice (animal model of dysferlinopathy) was shown to improve the skeletal muscle regeneration (Farini et al. 2012). Also dystrophic SCID/mdx mice developed much less diaphragm fibrosis than mdx mice (Farini et al. 2007). The same phenomenon was observed in nu/nu/mdx mice (Morrison et al. 2000). In our study, we compared SCID mice of the same genetic background as BALB/c mice. Thus, the fact that we noticed the higher level of fibrosis during regeneration of SCID mice muscles could be explained by the lack of T cells.

Immunodeficiency was previously shown to impact the histology of regenerating muscles. For example, more fibers with centrally positioned nuclei, i.e. not fully matured ones, were noticed in athymic BALB/c nude (nu/nu) mice than in C57BL/6 or BALB/c mice (Lagrota-Candido et al. 2010). Thus, the regeneration of BALB/c nude mice was delayed. Our studies showed that although T cell-mediated response was ablated in SCID mice, the muscle regeneration process was very effective and comparable to BALB/c mice. Injured SCID mouse muscles were completely reconstructed after 30 days. Importantly, the same number of cells was isolated from the intact muscles of both SCID and BALB/c. These cells constitute an almost pure population of satellite cells (Danoviz and Yablonka-Reuveni 2012). However, we noticed differences in the number of isolated cells and mononuclear cells present in the muscle at the early stages of regeneration. Nevertheless, these differences were not vital for the muscle reconstruction.

Our studies also showed that the lack of functional T cells led to the changes in macrophages contribution during muscles regeneration. In BALB/c mice the number of hematopoietic cells that were not macrophages, i.e. CD14−/CD45+, increased during first 24 h after the injury and these cells were detectable during subsequent 7 days of muscle regeneration. Next, M1 and M2 macrophages were identified in analyzed muscles based on the presence of CD68 or CD163 marker (Robertson et al. 1990; Rőszer 2015). Interestingly, BALB/c and SCID mice response to muscle injury was different at the level of macrophage involvement. M1 macrophages are characterized by a pro-inflammatory phenotype and play important role in promotion of T helper 1 (Th1) immune response (Rőszer 2015). M1 macrophages produce inflammatory cytokines and nitric oxide. Thus, participation of M1 macrophages in skeletal muscle regeneration was more prominent in SCID mice. Importantly, we were not able to detect anti-inflammatory M2 macrophages in regenerating SCID mice muscles. The polarization of M2 macrophages requires the presence of the Th2 immune response, that is absent in SCID mice. M2 macrophages play regulatory functions in tissue repair and remodeling (Rőszer 2015). They also promote of Th2-dependent immune response, have high phagocytotic capacity, synthesize extracellular matrix components, angiogenic and chemotactic factors (Rőszer 2015). Thus, we showed that SCID mice did not generate CD163 positive cells, i.e. M2, in the response to muscle injury. However, as we mentioned above, these differences were not translated into significant modifications in muscle regeneration.

Nevertheless, the immunodeficiency is an important factor considering cell transplantation experiments. It is essential for both xenogeneic and allogeneic transplants. The use of animals characterized by genetic deficiency is preferable to immune suppression. Two immunodeficient dystrophic models are used in transplantation studies, i.e. SCID/mdx and mdx/nude mice. Despite ablated T-cell-mediated responses in SCID and nude mice, functional natural killer (NK) cells are present. Thus, activity of NK cells must be suppressed using cyclophosphamide or specific antibodies (Brzoska et al. 2006). Another solution could be using SCID mice deficient for interleukin-2 receptor common gamma chain (IL2Rg), which are characterized with the ablation of NK cells, i.e. NOD/SCID;γ-c mice (NSG) (Traggiai et al. 2004). The NGS/mdx mice were described as mouse dystrophic model that can serve as xenogeneic transplants (Arpke et al. 2013). Summarizing, our study shows that even if the inflammation plays the crucial role in muscle regeneration in the absence of proper immunoresponse the skeletal muscle are able to effectively regenerate.
